# Identification of Interactive Genetic Loci Linked to Insulin Resistance in Metabolic Syndrome—An Update

**DOI:** 10.3390/medicina61010083

**Published:** 2025-01-07

**Authors:** Imadeldin Elfaki, Rashid Mir, Mohamed E. Elnageeb, Abdullah Hamadi, Zeyad M. Alharbi, Ruqaiah I. Bedaiwi, Jamsheed Javid, Tariq Alrasheed, Dalal Alatawi, Basmah M. Alrohaf, Mohammed K. Abunab, Turki Ahmed Muqri

**Affiliations:** 1 Department of Biochemistry, Faculty of Science, University of Tabuk, Tabuk 71491, Saudi Arabia; 2 Prince Fahd Bin Sultan Research Chair for Biomedical Research, Department of MLT, Faculty of Applied Medical Sciences, University of Tabuk, Tabuk 71491, Saudi Arabia; rashid@ut.edu.sa (R.M.); a.aldhafri@ut.edu.sa (A.H.); z.alharbi@ut.edu.sa (Z.M.A.); rbedaiwi@ut.edu.sa (R.I.B.); jali@ut.edu.sa (J.J.); 3 Department of Basic Medical Sciences, College of Applied Medical Sciences, University of Bisha, Bisha 61922, Saudi Arabia; melnageeb@ub.edu.sa; 4 Department of Internal Medicine, Faculty of Medicine, University of Tabuk, Tabuk 71491, Saudi Arabia; talrasheed@ut.edu.sa; 5 Haematology Laboratory, King Fahd Special Hospital, Ministry of Health, Tabuk 71491, Saudi Arabia; daaalatawi@moh.gov.sa; 6 Ministry of Health, Tabuk Region, Tabuk 71491, Saudi Arabia; bmalenzi@moh.gov.sa; 7 Saudi Ministry of Health, Umluj Region, Tabuk 71491, Saudi Arabia; mkabunab@gmail.com; 8 Asir Health Cluster, Tarj General Hospital, Bisha 67721, Saudi Arabia; Tmuqri@moh.gov.sa

**Keywords:** metabolic syndrome, insulin resistance, type 2 diabetes (T2D), cardiovascular disease (CDV), polycystic ovarian syndrome (PCOS), nonalcoholic fatty liver disease (NAFLD), genetic testing

## Abstract

Metabolic syndrome is a metabolic disorder characterized by hypertension, dyslipidemia, impaired glucose tolerance, and abdominal obesity. Impaired insulin action or insulin resistance initiates metabolic syndrome. The prevalence of insulin resistance is increasing all over the world. Insulin resistance results in the defective metabolism of carbohydrates and lipids, in addition to low-grade chronic inflammation. Insulin resistance is associated with metabolic syndrome, which is a risk factor for a number of pathological conditions, such as Type 2 diabetes (T2D), cardiovascular disease (CVD), nonalcoholic fatty liver disease (NAFLD), and polycystic ovarian syndrome (PCOS). Genome-wide association studies have increased our understanding of many loci linked to these diseases and others. In this review, we discuss insulin resistance and its contribution to metabolic syndrome and these diseases. We also discuss the genetic loci associated with them. Genetic testing is invaluable in the identification and stratification of susceptible populations and/or individuals. After susceptible individuals and/or populations have been identified via genetic testing or screening, lifestyle modifications such as regular exercise, weight loss, a healthy diet, and smoking cessation can reduce or prevent metabolic syndrome and its associated pathologies.

## 1. Introduction

**Figure 1:**
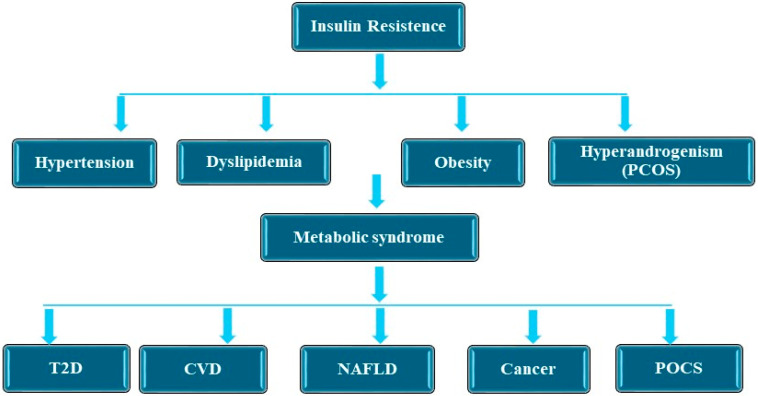
Metabolic Syndrome and associated diseases. Metabolic syndrome is associated with insulin resistance, and it is a risk factor for type 2 diabetes (T2D), cardiovascular disease (CVD), nonalcoholic fatty liver disease (NAFLD), polycystic ovarian syndrome PCOS), and cancer.

**Figure 2:**
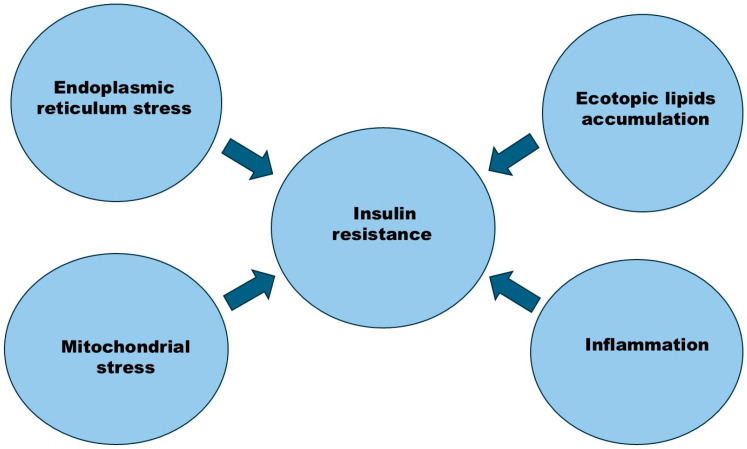
The factors that cause insulin resistance. Insulin resistance is caused by inflammation, mitochondrial stress, endoplasmic reticulum stress, and ectopic lipid accumulation.

The prevalence of metabolic syndrome ranges from 12.5 to 31.4% across the world [1]. Its prevalence has significantly increased in the Eastern Mediterranean and in America, and positively correlates with these countries’ level of income [1]. Metabolic syndrome is a complex condition defined by a combination of metabolic irregularities, including hypertension, atherogenic dyslipidemia, poor glucose tolerance, and abdominal obesity [2,3,4,5]. Atherogenic dyslipidemia is characterized by elevated triglyceride levels in the blood, reduced high-density lipoprotein (HDL), and normal or increased low-density lipoprotein cholesterol (LDL) [5]. This promotes the mobilization of free fatty acids from the adipose tissue of the abdominal region or central obesity [5]. It constitutes a risk factor for type 2 diabetes, atherosclerotic cardiovascular disease and nonalcoholic fatty liver disease (NAFLD), cancer, and polycystic ovarian syndrome (PCOS) (Figure 1) [3,6,7]. In addition, insulin resistance has been reported in type 1 diabetes with chronic kidney disease [8,9]. Insulin resistance is defined as a condition of decreased insulin sensitivity in the liver, skeletal muscles, and adipose tissues. In an insulin-resistant state, the tissues cannot effectively use the insulin for intracellular insulin-mediated glucose uptake, leading to insulin receptor desensitization [8]. In order to compensate for insulin resistance or insensitivity, the pancreatic beta cells secrete more insulin (hyperinsulinemia) to maintain sugar homeostasis [8]. This hyperinsulinemia results in dyslipidemia, hyperglycemia, and hypertension [8,10]. Insulin resistance is fundamental to its pathogenesis, impairing the proper metabolism of carbohydrates and lipids and fostering a condition of low-grade chronic inflammation (Figure 2) [11]. Understanding the complex interplay between insulin resistance and these illnesses is essential for formulating effective preventative and therapeutic measures. Recent advancements in genetic research have clarified the significance of particular genetic risk loci in the onset of metabolic syndrome, its related disorders, and other diseases. Genetic testing has become an essential tool for identifying at-risk individuals or populations, facilitating targeted interventions [12,13,14]. The genetic screening used to predict individuals at risk of T2D has been reported to have limitations, such as the small effect size of loci and the lack of suitable models for investigating gene–gene and gene–environment interactions in the prediction of T2D risk [15]. Nevertheless, it could be used in the future; for example, genetic studies will assist in stratifying individuals at risk of diabetes, and novel sequencing technologies will detect low-frequency and rare gene variants with large influence sizes [15]. The current genetic tests used to detect the risk of T2D include ACC2, which plays a key role in the synthesis of fatty acid and oxidation pathways [16], GCKR, which regulates glucose metabolism in the liver [17], and *HNF4A*, which encodes a transcription factor necessary for the development of the pancreas and hepatocytes [18]. Meanwhile, the current genetic tests used for the detection of CVD risk include ANGPTL4, which is involved in cholesterol metabolism [19], APOB, which is associated with dyslipidemia [20], and NOS3, which is important for vascular homeostasis [21]. In addition, the PNPLA3 and *TRIB1* genes are currently being used for genetic testing for the risk of NAFLD. PNPLA3 balances the storage and secretion of liver triglyceride [22], and (*TRIB1*) regulates liver lipogenesis [23]. Obesity is a risk factor for comorbid conditions such as CVD, T2D, cancer, asthma, osteoarthritis, chronic backache, obstructive sleep apnea, NAFLD, and diseases of the gallbladder [24]. Lifestyle adjustments, including consistent physical activity, weight control, nutritious dietary selections, and smoking cessation, have demonstrated efficiency in alleviating the effects of metabolic syndrome and its associated conditions [25]. Furthermore, bariatric surgery has been described as an effective treatment to reduce obesity and its associated comorbidities. However, bariatric surgery is associated with complications such as osteoporosis, nutritional or iron deficiency, anemia, and diarrhea [24,26].

In this review, we discuss insulin resistance and its associated pathological conditions; we also discuss the genetic loci associated with metabolic syndrome, as this condition can be detected earlier by genetic testing or screening for prevention and treatment strategies for the susceptible individuals or populations.

## 2. Insulin Resistance

The prevalence of insulin resistance is more than 40% in adults globally [27]. Insulin resistance is a precursor pathological condition of many metabolic diseases, and it is a condition of the decreased responsiveness of insulin-targeting tissues (liver, adipose tissues, and skeletal muscles) to physiological insulin levels [6]. Under physiologic conditions, the insulin-signaling pathway is a complex process with various enzymes and mediators resulting in insulin-mediated glucose uptake by adipose and muscular tissues. The pathway starts when insulin binds the alpha chain of the insulin receptor [28]. The insulin–insulin receptor complex results in structural changes to the beta chain by the auto-phosphorylation of the tyrosine residues [28]. This auto-phosphorylation recruits different proteins for phosphorylation to start the insulin signaling pathways, for example the insulin receptor substrates (IRSs), SHC-transforming (Shc) protein, and adapter protein with a PH and SH2 domain [29]. Thereafter, the IRSs are activated by kinases such as extracellular signal-regulated kinase 1/2), atypical protein kinase C, serine/threonine-protein kinase 2, Akt (protein kinase B), mTOR (mammalian target of rapamycin), and ROCK1 (rho-associated protein kinase 1 [29,30]. Then, the IRS-1 complexes activate the phosphoinositide 3-kinase (PI3K). Thereafter, the phosphatidylinositol 4, 5-bisphosphate (PIP_2_) is converted to phosphatidylinositol 3, 4, 5-trisphosphate (PIP_3_) [29,31]. The PIP_3_ activates Akt, and then, the activated Akt promotes cellular glucose uptake by the GLUT-4 localization. In addition, the activated Akt enhances glycogen synthesis via inhibition of the glycogen synthase kinase [29,31].

The mitogen-activated protein kinase pathway (MAPK) is also influenced by the insulin-signaling pathway [32]. The MAPK is implicated in the expression and translocation of genes and cell growth. Insulin is important in the regulation of carbohydrates, lipids, and protein metabolism [32]. A disturbance in the insulin-signaling pathway results in impaired insulin action and insulin resistance [33]. The dysfunction of mitochondria, stress of endoplasmic reticulum, oxidative stress and inflammation, lipotoxicity, hypoxia, and altered metabolomes are proposed causes of insulin resistance [8,34,35]. Oxidative stress is suggested to cause insulin resistance by inducing pancreatic beta cell dysfunction and affecting insulin secretion or production [36]. Another way for oxidative stress to develop insulin resistance is by affecting the expression and/or localization of GLUT4 [29]. In addition, oxidative stress is proposed to negatively affect the insulin-signaling pathway by affecting insulin receptor substrates 1 and 2 and the PI3K enzyme or by downregulation of the element of this pathway [29]. The factors of inflammation such as immune cells, acute-phase proteins, cytokines, adipokines, macrophage migration inhibitory, and blood coagulation play roles in insulin resistance as well [8,37]. These factors of inflammation activate the intracellular serine/threonine kinases inhibitors and thereby inhibit important proteins of the insulin-signaling pathway leading to insulin resistance [8,38]. Cytokines and inflammatory mediators, for example, tumor necrosis factor alpha and nuclear factor kappa b, stimulate the Janus kinase pathways, leading to the phosphorylation of serine of IRS-1, further leading to impaired insulin action [29]. Insulin resistance followed by the pathologic elevation of blood sugar levels are the important clinical symptoms of T2D [39]. In the state of pre-prediabetes, the levels of insulin rise meet the physiological needs of insulin resulting in chronic hyperinsulinemia, with increased blood sugar leading to pancreatic beta cell failure and T2D development [6,40]. The accumulation of fats in the myocytes has been proposed as the cause of insulin resistance in skeletal muscles. This is probably because of the high oxidation of fatty acids and reduced insulin-induced utilization of glucose in muscle tissues [11,41]. In insulin-resistant young individuals, there is a decreased activity of mitochondrial phosphorylation and oxidation in skeletal muscles [11]. This reduced mitochondrial activity is probably due to the decreased density of mitochondria in skeletal muscles [11]. The reduced density of mitochondria may be caused by the decreased expression of the lipoprotein lipase (LpL) gene in skeletal muscle [11]. This results in the decreased peroxisome proliferator-activated receptor delta (PPARδ)-mediated biogenesis of mitochondria [11,42]. The accumulation of fats (e.g., diacylglycerol and ceramides) is associated with insulin resistance in the skeletal muscles (Figure 2). It has been reported that diacylglycerol accumulation leads to impairment of the insulin signaling in the insulin-stimulated phosphorylation of tyrosine residue of insulin receptor substrate-1 (IRS-1) and IRS-1–associated activation of the phosphatidylinositol 3 kinase in the skeletal muscle [6,11]. The accumulation of ceramides and diacylglycerols (DAG), the activation of the protein Kinase C-epsilon results (PKCε), and the loss of the insulin-mediated activation of Akt lead to the impairment of the liver insulin signaling pathway [11,43]. PKCε catalyzes the phosphorylation and the inhibition of the insulin receptor [43]. The serine/threonine kinase Akt, also called the protein kinase B (PKB), is an important element in the metabolism of carbohydrates and lipids [44]. The de novo liver lipid synthesis is important for the regulation of the triacylglycerol content in hepatocytes [45]. Hyperglycemia and hyperinsulinemia promote de novo liver lipid synthesis in individuals with NAFLD. Loss of weight reduces the triacylglycerol in liver cells [45,46].

In obese individuals, the defective adipose tissues play important roles in the induction of insulin resistance [47]. The mechanism through which obese adipose tissues develop insulin resistance is not fully understood [47]. The insulin resistance in adipose prompts the adipose tissue to secrete excessive free fatty acids into the blood stream. This enhances the synthesis of diacylglycerol (DAG) and triacylglycerol (TAG) in hepatocytes and myocytes leading to ectopic deposition of fats [48]. DAG activates the protein kinase C (PKCθ) theta isoform in myocytes and PKC (PKCε) epsilon isoform in the hepatocytes [48]. This results in the impairment of the insulin signaling pathway and insulin resistance in liver and muscles. This also leads to systemic insulin and different metabolic disturbances such as hyperglycemia, hypertension, increased blood lipids, and NAFLD [48]. Adipose tissue insulin resistance is associated with metabolic syndrome, and it represents an important predictor for this syndrome [48]. The brown adipose tissue plays an important role in the regulation of energy and sugar homeostasis and is associated with levels of glucose and insulin resistance in peripheral tissues [49], whereas the white adipose tissue (visceral adipose tissues surrounding the abdominal region) is the main source of the inflammatory markers in T2D, as it secretes the cytokines (tumor necrosis factor-alpha and interleukins 1, 6, 10), adipokines, chemokines, plasminogen activator inhibitor-1, and immune cells such monocyte chemoattractant protein [49,50]. These white adipose tissues also are the source of immune cells, macrophages, and T and B-lymphocytes. These inflammatory elements induce chronic inflammation, insulin resistance, and T2D [49].

In addition, blood leptin (a hormone produced by the adipose tissues) levels are negatively correlated with insulin sensitivity [51]. In a study conducted in mice, it was reported that a quantitative trait locus, localized on chromosome 14, is associated with hyperinsulinemia, and a region of uncoupling protein 2 and 3 locus on chromosome 7 is associated with hyperleptinemia. The interaction of these loci develops insulin resistance and increases the blood levels of glucose and leptin [52].

## 3. Diseases Associated with Metabolic Syndrome

### 3.1. Type 2 Diabetes (T2D)

T2D is a heterogeneous disorder characterized by hyperglycemia and caused by multiple pathophysiologic pathways with contributions of insulin insensitivity or resistance and pancreatic beta cell dysfunction among subgroups and individuals [53]. T2D cases can range from those with a phenotype with insulin resistance but having some functioning pancreatic beta cells to a phenotype that requires early insulin therapy [54,55]. T2D is one of the most common metabolic diseases. It is characterized by increased blood sugar and has a very important socioeconomic impact due to its very serious complications [39]. These complications are microvascular and macrovascular [56]. The microvascular complications of T2D are diabetic nephropathy, retinopathy, and neuropathy [56]. Diabetic retinopathy and nephropathy result in the gradual deterioration of blood vessels in renal tissues and retina leading to end-stage kidney disease and blindness, respectively [56]. Diabetic neuropathy negatively influences the nerves in the peripheral tissues leading to pain, muscular weakness, impairments of sensory function, and possible amputation of limbs [57]. The macrovascular complications of diabetes include atherosclerosis, coronary heart disease (CHD), cerebrovascular disease, and peripheral vascular or arterial disease [58]. The risk factors of T2D are genetic and environmental [59]. The genome-wide association studies and exome and genome-wide sequencing methods have broadened our current understanding of the genetic loci associated with the risk to T2D [60,61,62,63,64,65,66]. The genetic loci associated with the risk to T2D (Table 1) include the genes implicated in the secretion of insulin, for example the KCNJ11 gene [64,67]. Moreover, the *PPARG* gene that influences energy balance is suggested as a risk locus for obesity and defective insulin signaling [64].

**Figure 3:**
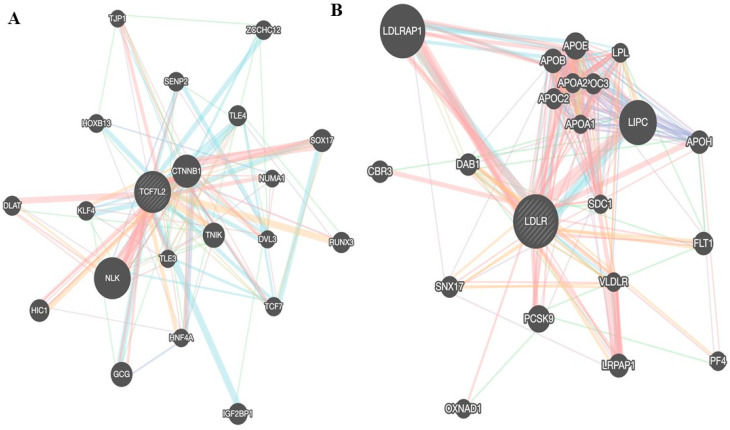

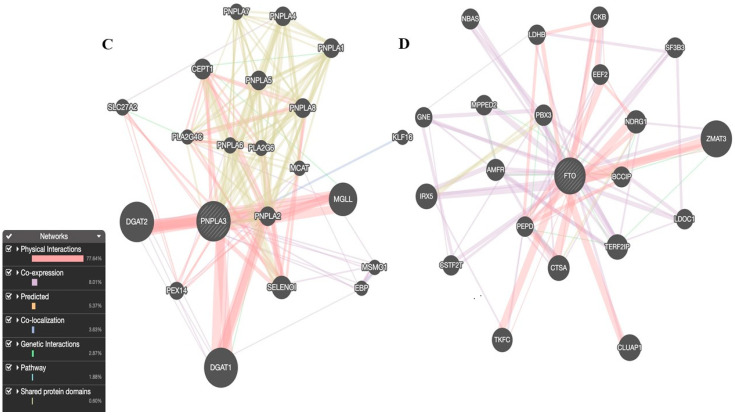
Gene interaction network of certain genes associated with metabolic syndrome comorbidities. The interaction network of (**A**) TCFL2, which is associated with T2D, (**B**) LDLR associated with CVD, (**C**) PNPLA3 associated with NAFLD, and (**D**) the FTO gene associated with PCOS. This figure was prepared using GENEMANIA.

In addition to the genes involved in insulin signaling for instance glucose transporters [68,69], other loci involved in insulin resistance (Figure 3) and T2D include the genes *BCL2*, *FAM19A2*, *CCND2*, *PAM*, *PDX1*, and *Cyp450* [64,66,70,71]. The environmental risk factors of T2D include increased body weight or obesity, unhealthy diet, and decreased physical activity [72]. T2D is induced by pancreatic beta cell dysfunction, i.e., the secretion of insulin is decreased resulting in hyperglycemia. In addition, insulin resistance develops in peripheral tissues [39,73]. In an excessive food intake as in an overweight condition, elevated blood sugar and elevated blood lipids lead to chronic inflammation and insulin resistance [39]. Under these conditions, the pancreatic beta cells are exposed to toxic pressures of inflammation [49] and different stresses, for example, endoplasmic reticulum (Figure 2) [74], metabolic, oxidative, and amyloid [75]. These stresses may result in pancreatic beta cells’ dysfunction [75]. The pancreatic beta cells’ failure was proposed due to beta cell inflammation in individuals with a genetic predisposition, due to the stress of these cells. The stress in beta cells is caused by increased insulin secretion to compensate for peripheral insulin resistance. In the local inflammation in pancreatic beta cells, the macrophages secrete IL-1 and IL-1beta, which induce beta cell apoptosis. In addition, oxidative stress may also produce reactive oxygen species (ROS) that contribute to beta cell dysfunction [49]. Moreover, the elevated plasma levels of C-reactive protein (CRP) and other pro-inflammatory cytokines such as interleukin-6 (L-6) and Tumor necrosis Factor-alpha (TNFα) were reported to induce pancreatic beta cell atrophy and dysfunction in type 2 diabetic rats [76]. It has been reported that lifestyle intervention including physical activity, weight loss, and diet modification can delay or prevent T2D [77].

**Table 1:** The single nucleotide variations associated with metabolic syndrome, T2D, CVD, NAFLD, and PCOS.

Metabolic Syndrome	Chromosome	Locus	SNP	Effect	Reference
	12	Lrp1	rs4759277 C/A	Insulin resistance	Delgado et al. [78]
	02	IRS1	rs2943634 A/C	Associated with HbA1C, triglycerides, and HDL cholesterol	Povel et al. [79]
	16	CETP	rs5882 A/G	Associated with low HDL cholesterol and high blood pressure	Esfandiar et al. [80]
	11	BUD13	rs10790162 A/G	Associated with lipids and carbohydrate metabolism	Kang et al. [81]
	11	ZPR1	rs2075290 C/T	Associated with lipids and carbohydrate metabolism	Kang et al. [81]
	22	MKL1	rs4507196 C/A	Associated with lipids and carbohydrate metabolism	Kang et al. [81]
T2D	Chromosome	Locus	SNP	Effect	Reference
	07	JAZF1	rs864745 A/G	Impaired insulin signaling	Alharbi et al. and Vujkovic et al. [82,83]
	03	ADCY5	rs11708067 A/G	Defective insulin secretion	Vujkovic et al. and Roman et al. [83,84]
	10	TCF7L2	rs7903146 C/T	Defective insulin secretion and impaired liver insulin sensitivity	Cropano et al. [85]
	3	PPARG	rs1801282 C/G	Promotion of insulin signaling and decreased T2D risk	Sarhangi et al. [86]
	3	IGF2BP2	rs4402960 G/T	Beta cell dysfunction	Nfor et al. [87]
	8	SLC30A8	rs13266634 C/T	Impaired insulin signaling	Dong et al. [88]
CVD	Chromosome	Locus	SNP	Effect	Reference
	9	CDKN2A/B	rs4977574 G/A	Atherosclerosis and proliferation of vascular smooth muscles	Li et al. [89]
	1	NOS3	rs1799983 G/T	Endothelial dysfunction	Luo et al. [90]
	19	APOE	rs7412 C/T	Hypercholesterolemia	Semaev et al. [91]
	6	MTHFR	rs1801133 C/T	Elevated homocysteine	Luo et al. [92]
	9	ABO	rs8176746 T/C	Hypercholesterolemia	Paquette et al. [93]
	19	LDL-R	rs688 C/T	Hypercholesterolemia	Dai et al. [94]
NAFLD	Chromosome	Locus	SNP	Effect	Reference
	22	PNPLA3	rs738409 C/G	Increased hepatic fat levels and inflammation	Romeo et al. [95]
	19	TM6SF2	rs58542926 C/T	Abnormal LDL-cholesterol secretion	Kozlitina et al. [96]
	2	GCKR	rs1260326 C/T	Impaired hepatic glucose and lipid metabolism	Nisar et al. [97]
	1	FNDC5	rs3480 A/G	Causes steatosis	Dwi Astarini et al. [98]
	19	MBOAT7	rs641738 C/T	Probably influences glucose metabolism by modulating fat liver content	Umano et al. [99]
PCOS	Chromosome	Locus	SNP	Effect	Reference
	2	IRS1	rs1801278 A/G	Insulin resistance	Gonzalez et al. [100]
	19	VDR Apa I	rs7975232A/C	Causes anti-Müllerian hormone and insulin resistance	Pei et al. and Siddamalla et al. [101,102]
	17	SLC6A4 5HTTLPR	rs25531 A/G	Influences the secretion of insulin	Senk et al. [103]
	11	CYP2R1	rs2060793 A/G	Influences Vitamin D synthesis or activation	Haldar et al. [104]
	6	TNF-α	rs361525 A/G	Defective immune response	Boots et al. and Alkhuriji et al. [105,106]
	3	ADIPOQ	rs1501299 A/C	Causes obesity and insulin resistance	Alfaqih et al. [107,108]
	16	FTO	rs1421085C/T	Obesity and increased BMI	Song et al. [109]

### 3.2. Cardiovascular Disease (CVD)

Cardiovascular diseases (CVDs) are very important cause of deaths and disabilities all over the world. The risk factors for CVDs are unhealthy nutrition, dyslipidemia, elevated blood pressure and blood sugar, being overweight, smoking and physical inactivity, gender differences, alcohol addiction, older age, and race [110]. CVDs include ischemic heart disease or pulmonary embolism, thromboembolism, coronary heart disease and cerebrovascular disease, peripheral vascular or arterial disease leading to myocardial infarction, and stroke [111]. These disorders are mostly complications of atherosclerosis [112]. Atherosclerosis is the accumulation of lipids and fibrous and calcified tissues in the intima of arteries with activation of the vascular endothelial cells and inflammatory reactions resulting in the formation of atheroma plaques [112]. Low-density lipoprotein cholesterol (LDL-C) is retained, accumulated, and modified in the intima of the blood vessels and initiates vascular endothelial dysfunction. The accumulation of the modified LDL-C with atherogenic factors (products of cholesterol oxidation, aldehydes, saturated fatty acids lauric, and myristic acid) enhances endothelial cell activation and the recruitment of macrophages. These macrophages uptake the modified LDL-C and excess lipids resulting in the generation of foam cells [112,113]. Then, vascular smooth muscle cells move to the arterial intima and produce a fibrous cap. This cap stabilizes the atherosclerotic plaque, while the increased accumulation of the foam cells results in necrosis of the plaques from inside [113]. Then, the necrotic part increases, the fibrous cap decreases, and the necrotic part interacts with blood clotting factors and cells leading to induction of thrombosis [113]. In the case of obesity, the increased fat is stored in white adipose tissues. Then, the adipocytes become enlarged and develop insulin resistance with hyperlipolysis [50]. Insulin resistance was reported to cause a lipid triad, first, increased blood triglyceride, second, decreased levels of high-density lipoprotein–cholesterol, and third, the presence of small dense low-density lipoproteins, in addition to excessive postprandial hyperlipidemia. The hyperlipidemia increases the susceptibility to CVD [50,114,115]. The defective vasodilatation, abnormal flow of blood, and impaired renin–angiotensin–aldosterone system (RAAS) are possible causes of the association between hypertension and insulin resistance. The RAAS and increased blood insulin activate the MAPK pathway [116]. This has a deleterious effect on the blood vessel walls leading to endothelial dysfunction and atherosclerosis [116]. Moreover, in an insulin-resistant condition, there is imbalance of vascular dilatation and vascular constriction since insulin regulates nitric oxide (vasodilator) and endothelin-1 (vasoconstrictor) [50]. This imbalance results in hypertension, endothelial dysfunction, and atherosclerosis [50]. In addition, inflammation was also reported to induce insulin resistance in cardiovascular disease. The increased lipids induce inflammatory response and signaling pathways such as c-Jun N-terminal kinase (JNK), IkappaB kinase, and nuclear factor Kappa B (NF-kB), leading to the secretion of cytokines such as TNF-alpha, interleukin-6, and 1 beta [50]. Genetic risk loci for CVD include the low-density lipoprotein cholesterol receptor (LDLR) (Figure 3) or proprotein convertase subtilisin/kexin type 9 (PCSK9), as they result in hypercholesterolemia, which is a traditional risk factor for CVD [117,118]. The CVDs can be delayed or prevented by diet modification, regular exercise, reducing weight, no smoking and no alcohol, lipids lowering treatment, and blood pressure control [119].

### 3.3. Polycystic Ovarian Syndrome (PCOS)

More than 40% of adult females and about 30% of teenage females with PCOS suffer from metabolic syndrome [120]. The clinical characteristics of PCOS include insulin resistance, increased body weight, dyslipidemia, and hyperandrogenism [121]. Therefore, PCOS can be classified as a metabolic syndrome [120]. Insulin resistance and compensatory hyperinsulinemia were reported in a large percentage of PCOS patients. The main mechanism of insulin receptor abnormality leading to IR is the defective post-binding of insulin to its receptor, and because of the increased phosphorylation of serine and the reduced phosphorylation of tyrosine, it results in the reduced insulin activation of the phosphatidylinositol-3-kinase (PI3k) signaling pathway that activates the transportation of glucose [120,122]. Moreover, microRNA abnormality is reported in PCOS. For example, it has been suggested that mir-122 suppresses the expression of insulin-like growth factor 1 leading to insulin resistance [120,123]. In addition, insulin resistance was also suggested from the elevated high mobility group box 1 (HMGB1) results in the development of insulin resistance in granulosa cells of PCOS subjects [124]. This elevated HMGB1 is linked to the deterioration of autophagy by HMGB1 [124]. It has been recommended that the control of HMGB1 production is probably useful to ameliorate insulin resistance in granulosa cells of PCOS patients [124]. Moreover, in PCOS there is disturbance in the flora of the intestine causing dysbiosis, which contributes to insulin resistance development by mechanisms like endotoxemia, certain peptides of the gut–brain, abnormal metabolites, and hyperandrogenism [120,125]. The mitochondrial dysfunction, endoplasmic reticulum, and oxidative stress are also implicated insulin resistance in PCOS [120,126,127]. In addition, hyperandrogenism results in the accumulation of fats in adipose tissue, particularly around the abdominal region, leading to insulin resistance [120,128]. Another suggested mechanism of hyperandrogenism in the development of PCOS is dihydrotestosterone (DHT), which may be implicated in the mitochondrial fission of granulosa cells and thus negatively affects ovulation; hyperandrogenism may also result in endoplasmic reticulum stress, leading to oocyte damage [129,130]. Furthermore, hyperandrogenism probably induces low-grade chronic ovaritis producing the NOD-, LRR-, and pyrin domain-containing protein 3 inflammasome, leading to the pyroptotic death of granulosa cells, ovarian dysfunction, and fibrosis [131]. The genetic loci associated with PCOS risk (Figure 3) include the CYP11a gene that encodes the cytochrome p450 enzyme, which catalyzes an intermediate step in the conversion of cholesterol to progesterone [132]. In addition, the Luteinizing hormone (LH) and the LH receptor gene variations were reported to be associated with susceptibility to PCOS [133]. Methods of preventing PCOS include a good diet pattern and regular physical activity; it is important to give attention to the psychological assessment of PCOS management and restoration of gut microbiota by probiotics, prebiotics, or a fecal microbiota transplant [134,135].

### 3.4. Nonalcoholic Fatty Liver Disease (NAFLD)

Metabolic syndrome can cause NAFLD, and insulin resistance is associated with metabolic syndrome [136]. The NAFLD is a chronic liver disease and is defined as the existence of fat in the hepatocytes (˃5–10% of hepatocytes are fatty) with no other known causes of steatosis, for example alcohol abuse, viral infection, drugs, toxins, autoimmune disease, or overload of iron [136]. NAFLD develops from the interaction of environmental, behavioral, and genetic risk factors [137], where the environmental and behavioral risk factors include smoking, air pollution, an unhealthy diet, and physical inactivity [137,138]. NAFLD varies from the simple infiltration of fats, with no inflammation (known as non-alcoholic fatty liver (NAFL)), to fat infiltration with inflammation (known as non-alcoholic steatohepatitis (NASH)), liver fibrosis, and liver cirrhosis, which can develop into end-stage liver disease (ESLD) or to hepatocellular carcinoma (HCC) [136,139]. Complications of NAFLD include CVD and chronic kidney disease (CKD) [140]. Factors leading to NAFLD include insulin resistance, excessive lipid accumulation and increased activation of lipid signaling resulting in cellular distress, the activation of natural immunity, a microbiome with genetic susceptibility (PNPLA3 gene), and an unhealthy diet (saturated fatty acids and fructose) and physical inactivity [140]. Certain cases with NAFL showed a lipodystrophic phenotype in which there is a defective storage of subcutaneous fats and increased liver fat storage associated with elevated insulin resistance [141,142]. Genetic loci associated with NAFLD include PPARγ, c-fos, p85α, Phosphate Cytidylyltransferase 1 Alpha, Patatin-Like Phospholipase Domain-Containing Protein 3 (PNPLA3), and WRN [142]. The PNPLA3 gene encodes an enzyme found fat tissues, retina, skin, and the hepatocytes [137,143]. In the liver, the PNPLA3 exists in hepatocytes and hepatic stellate cells, and it is involved in regulation of lipids and retinol metabolism [144,145]. The PNPLA3 level is increased in a fed state and is regulated by insulin [143]. A gene variation (missense mutation, I148M) of the PNPLA3 is associated with susceptibility to NAFLD [137,144]. In addition, the Transmembrane 6 Superfamily Member 2 (TM6SF2) was also reported as a risk locus for the NAFLD [146]. The TM6F2 gene encodes TM6F2 protein that is mainly present in the hepatocytes and small intestine. In the liver, TM6SF2 regulates the metabolism of lipids, implicated in the regulation of TG-rich lipoprotein synthesis and secretion, and influences lipid droplet content [146]. The Membrane Bound O-Acetyltransferase Domain Containing 7 (MBOAT7) is also reported as a risk locus for NAFLD. This gene encodes the lysophosphatidylinositol (LPI) acyltransferase and is also involved in lipid metabolism in liver cells [147]. Furthermore, the Glucokinase Regulator (GCKR) gene is also a risk locus for NAFLD. This gene encodes a protein that allosterically inhibits glucokinase (GCK), a very important glucose homeostatic enzyme [147,148,149].

In fact, there are certain loci associated with metabolic syndrome and the related traits and diseases. For example, the IRS1 (insulin receptor substrate 1) gene was reported to be associated with insulin resistance, T2D [150,151], and PCOS [152]. In addition, the transcription factor 7-like 2 (TCF7L2) was reported to be associated with hyperlipidemia, CVD [153], T2D [154], and PCOS [155]. Furthermore, CHI3L1 (Chitinase-3-like protein 1) and CD36 are associated with associated with insulin resistance, T2D, and CVD [156]. LEPR (leptin receptor) resistin (RETN) is associated with insulin resistance, T2D, CVD, and PCOS [156,157,158]. Moreover, the *FTO* gene is linked to obesity, NAFLD, and T2D [159]. In addition, *SLC2A2* gene plays a role in insulin resistance, T2D, and PCOS development [160].

## 4. Conclusions and Future Perspectives

Genome-wide association studies (GWASs) helped to understand the genetic loci associated with metabolic syndrome and hence can be utilized for the detection and identification of susceptible individuals or populations via genetic testing or screening for prevention of this pathological condition and its comorbidities. Genetic testing has become available and is a reliable tool for the prediction of T2D risk for individuals with or without awareness of their risk of T2D [161]. Genetic testing for CVD also provides polygenic risk scoring and gives healthcare providers new opportunities for personalized CVD risk prediction and prevention [13]. Moreover, the inclusion of genetic testing for PCOS and NAFLD assists in risk assessment.

## 5. Conclusions

Metabolic syndrome is a metabolic disorder characterized by obesity, hypertension, disturbed blood lipid profile, glucose intolerance, elevated blood pressure, impaired glucose tolerance, hyperinsulinemia, and impaired insulin action or insulin resistance. The metabolic syndrome prevalence is predicted to increase with the current global increase in obesity [162]. Therefore, the identification of individuals at high risk of developing metabolic syndrome and its associated comorbidities is very important for researchers and health care providers [15]. Metabolic syndrome a predisposing factor for a number of pathological conditions including T2D, CVD, PCOS, and NAFLD. Metabolic syndrome can be prevented by lifestyle modifications such as regular physical exercise, healthy diet, no smoking, no alcohol, and weight loss.
